# Clinical and molecular analysis in a cohort of Chinese children with Cornelia de Lange syndrome

**DOI:** 10.1038/s41598-020-78205-5

**Published:** 2020-12-04

**Authors:** Qun Li, Guoying Chang, Lei Yin, Juan Li, Xiaodong Huang, Yongnian Shen, Guoqiang Li, Yufei Xu, Jian Wang, Xiumin Wang

**Affiliations:** 1 Department of Endocrinology and Metabolism, Shanghai Children’s Medical Center, Shanghai Jiaotong University School of Medicine, 1678 Dongfang Road, Shanghai, 200127 China; 2 Department of Rare Disease Clinic, Shanghai Children’s Medical Center, Shanghai Jiaotong University School of Medicine, Shanghai, China; 3 Department of Medical Genetics and Molecular Diagnostic Laboratory, Shanghai Children’s Medical Center, Shanghai Jiaotong University School of Medicine, Shanghai, China

**Keywords:** Diseases, Genotype

## Abstract

Cornelia de Lange Syndrome (CdLS) is a rare genetic disorder, which causes a range of physical, cognitive, and medical challenges. To retrospectively analyze the clinical characteristics and genetic variations of Chinese patients, and to provide experience for further diagnosis and treatment of CdLS in Chinese children, we identified 15 unrelated Chinese children who presented with unusual facial features, short stature, developmental delay, limb abnormalities, and a wide range of health conditions. In this study, targeted-next generation sequencing was used to screen for causal variants and the clinically relevant variants were subsequently verified using Sanger sequencing. DNA sequencing identified 15 genetic variations, including 11 *NIPBL* gene variants, two *SMC1A* gene variants, one *RAD21* gene variant, and one *HDAC8* variant. The phenotype of these patients was summarized and differences between this cohort and another four groups were compared. The clinical manifestations of the patients in this cohort were mostly consistent with other ethnicities, but several clinical features in our cohort had different frequencies compared with other groups. We identified 15 deleterious variants of which 11 were novel. Variants in the *NIPBL* gene were the most common cause in our cohort. Our study not only expands upon the spectrum of genetic variations in CdLS, but also broadens our understanding of the clinical features of CdLS.

## Introduction

Cornelia de Lange syndrome (CdLS, OMIM#122470, 300590, 300882, 610759, and 614701), which is also called Brachmann de Lange syndrome, is a multiple congenital anomaly syndrome characterized by typical facial features, growth impairment and intellectual disability, limb reduction defects, and involvement of other systems^1^. The prevalence of CdLS is estimated to be between 1 in 10,000 and 1 in 30,000 live births^2^. Although most cases of CdLS are dominant and sporadic, recurrence in siblings due to parental mosaicism has been reported^3^.

Genetic variations leading to CdLS have been identified in the following seven genes: *NIPBL*, *SMC1A*, *SMC3*, *RAD21*, *BRD4*, *HDAC8*, and *ANKRD11*^2^. Among these genes, *NIPBL* is a cohesion loading factor. *SMC1A*, *SMC3*, and *RAD21* code for structural components of the cohesin complex. *HDAC8* codes for an SMC3 deacetylase that is involved in cohesin recycling. Cohesin proteins are involved in regulating chromosome segregation, gene expression, DNA repair, and maintenance of genome stability^4^. Genotype–phenotype correlations have shown that *NIPBL* variants usually result in more severe phenotype than variants in other genes^5^. In addition, other genes were described in patients presenting features of CdLS or CdLS-like phenotype as well (i.e. *EP300*, *AFF4*)^2^.

In order to explore the clinical phenotype spectrum and gene spectrum of Chinese CdLS, we retrospectively analyze the clinical characteristics and genetic variations of our patients. In this study, we analyse the clinical features and the results of genetic testing of 15 patients who were diagnosed with CdLS. All of the patients harbored variants in *NIPBL*, *SMC1A*, *RAD21*, or *HDAC8*, of which 11 variants were novel. The phenotype of these Chinese patients was compared with four other groups.

## Materials and methods

### Patients

A total of 15 affected and unrelated children from Chinese families who were referred to Shanghai Children’s Medical Center were included in the study. Detailed medical history was recorded by pediatricians. Their clinical data were collected, such as sex, age, birth history, family history, clinical symptoms, craniofacial features, limb features, height, and weight, and laboratory examinations were performed. The preliminary diagnosis of CdLS was made by pediatricians and based on clinical manifestations and laboratory examinations according to the Diagnostic Criteria for Cornelia de Lange Syndrome by Antonie D. Kline (2007)^1^ and the clinical diagnostic criteria by the first international consensus statement (2018)^2^. Informed consent for the genetic analysis was obtained from the patients’ parents. This study was approved by the Ethics Committee of Shanghai Children’s Medical Center. All methods were performed in accordance with the relevant guidelines and regulations [International Ethical Guidelines for Health-related Research Involving Humans, Fourth Edition. Geneva. Council for International Organizations of Medical Sciences (CIOMS); 2016].

### Targeted next-generation sequencing and data analysis

Targeted next-generation sequencing (NGS) was performed as described in our previous study^6^. Briefly, both coding exons and flanking intronic regions were enriched using an XT Inherited Disease Panel (cat No.5190-7519, Agilent technologies Inc., Santa Clara, CA, USA) consisting of 2742 genes. *BRD4* and the other genes involved in CdLS-like phenotype are not included in this panel. Sequencing was performed on an Illumina HiSeq 2500 System (Illumina, San Diego, CA, USA). Alignment of the sequence reads to a reference human genome (Human 37.3; SNP135) was performed using NextGENe (SoftGenetics, State College, PA, USA). All single nucleotide variants (SNVs) were saved in a VCF format file and uploaded to Ingenuity Variant Analysis (Ingenuity Systems, Redwood City, CA, USA) for biological analysis and interpretation. The variants detected by NGS were validated by Sanger sequencing in the patients and their parents if the samples were available. According to the variant-interpretation guidelines from the American College of Medical Genetics and Genomics (ACMG) and the Association for Molecular Pathology^7^, which was evolved by ClinGen Sequence Variant Interpretation Working Group (https://www.clinicalgenome.org/working-groups/sequence-variant-interpretation/)^8,9^, we categorized the pathogenicity of variants.

### Statistics analysis

Statistical analysis for comparison between our cohort and four other groups was performed by (corrected) the chi-square test or Fisher’s exact test using SPSS 22.0 software. P values were adjusted by pairwise comparison using the Bonferroni test. P < 0.05 was considered statistically significant.

### Ethical approval

The Ethics Committee of Shanghai Children’s Medical Center approved the study (SCMCIRB-W2019014, 19 September 2019).

## Results

### Gene variants

**Figure 1:**
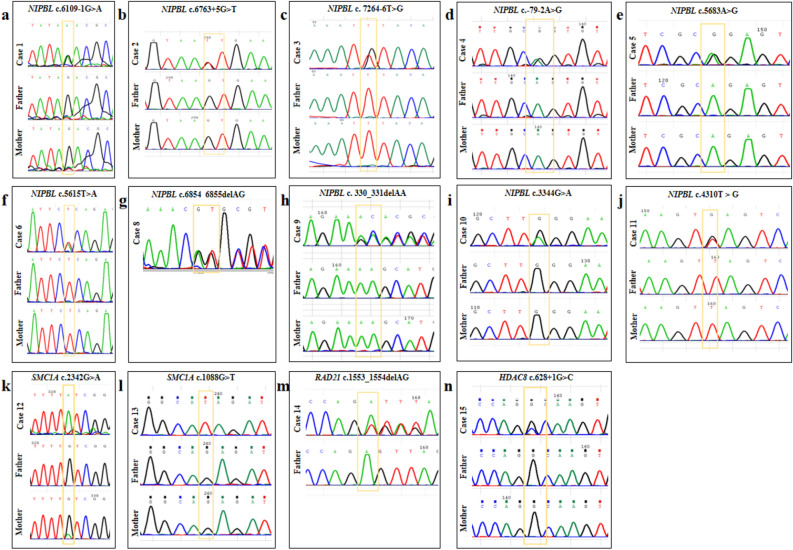
Variants identified in the P1-6 (**a**–**f**) and P8-15 (**g**–**n**).

A CdLS-related gene variant was found in all of the 15 patients, 14 were diagnosed by genetic testing in our hospital and one case was tested in another hospital and referred to our hospital for genetic counseling (Table 1). Among the 15 patients, 11 *NIPBL* gene variants (11/15, 73.3%; 4 splicing, 3 missense, 2 frameshift, and 2 nonsense variants), two *SMC1A* gene missense variants (2/15, 13.3%), one *RAD21* gene frameshift variant (1/15, 6.7%), and one *HDAC8* gene splicing variant (1/15, 6.7%) were detected. Of the 15 identified variants, 11 were novel, and p. (Cys781Phe) in *SMC1A* and c.6763 + 5G > T, c.7264-6 T > G, and c.-79-2A > G in *NIPBL* have been reported previously^10–13^. Except for patient 8 (P8) whose parents’ samples were not available and P14 whose mother’s sample was not available, the mother of P12 carried the same variant and the other 12 patients had de novo variants (Fig. 1). According to ACMG guidelines, 15 gene variants were classified, among which seven were pathogenic and eight were likely pathogenic (Supplementary Table S1).

**Table 1:** Variants identified in our patients.

Case	Variants	Location	Type	Homo/Het	Inherited or De novo	Novel or reported	ACMG classification
1	*NIPBL*(NM_133433.3) : c.6109-1G > A	Intron 34	Splicing	Het	De novo	Novel	P
2	*NIPBL* (NM_133433.3) : c.6763 + 5G > T	Intro 39	Splicing	Het	De novo	Reported	P
3	*NIPBL* (NM_133433.3) : c.7264-6T > G	Intron 42	Splicing	Het	De novo	Reported	LP
4	*NIPBL*(NM_133433.3) : c.-79-2A > G	5′UTR regions	Splicing	Het	De novo	Reported	LP
5	*NIPBL* (NM_133433.3) : c.5683A > G, p. (Arg1895Gly)	Exon 30	Missense	Het	De novo	Novel	P
6	*NIPBL* (NM_133433.3) : c.5615T > A, p. (Leu1872His)	Exon 30	Missense	Het	De novo	Novel	LP
7	*NIPBL*(NM_133433.3) : c. 6722T > C, p. (Leu2241Pro)	Exon 39	Missense	Het	De novo	Novel	LP
8	*NIPBL*(NM_133433.3) : c.6854_6855delAG, p. (Gln2285Argfs*3)	Exon 40	Frameshift	Het	NA	Novel	LP
9	*NIPBL* (NM_133433.3) : c.330_331delAA, p. (Ser111Hisfs*16)	Exon 4	Frameshift	Het	De novo	Novel	P
10	*NIPBL*(NM_133433.3) : c.3344G > A, p. (Trp1115*)	Exon 12	Nonsense	Het	De novo	Novel	P
11	*NIPBL* (NM_133433.3) : c.4310 T > G, p. (Leu1437*)	Exon 19	Nonsense	Het	De novo	Novel	P
12	*SMC1A* (NM_006306.2) : c.2342G > A, p. (Cys781Phe)	Exon 15	Missense	Hemizygote	Mother	reported	LP
13	*SMC1A*(NM_006306.2) : c.1088G > T, p. (Arg363Ile)	Exon 6	Missense	Hemizygote	De novo	Novel	LP
14	*RAD21* (NM_006265.2) : c.1553_1554delAG, p. (Glu518Valfs*18)	Exon 12	Frameshift	Het	NA	Novel	LP
15	*HDAC8*(NM_018486.2) : c.628 + 1G > C	Intron 6	Splicing	Het	De novo	Novel	P

*Homo* homozygosis, *Het* heterozygosis, *NA* not available, *LP* likely pathogenic, *P* pathogenic.

### Clinical manifestations

A total of 15 patients were included in the study, including nine (60.0%) boys and six (40.0%) girls. The patients ranged in age from 3 months to 10 years and 2 months, with a median age of 4 years. They underwent a comprehensive clinical evaluation. Detailed data of the evaluation are shown in supplementary Table S2. The patients were scored using clinical diagnostic criteria^2^ and 10 (66.7%) scored > 11 points.

Among the typical facial features of CdLS, except for P15, the other 14 patients showed long eyelashes. Thirteen patients had thick eyebrows and arched eyebrows except P14 and P15. Additionally, microcephaly (93.3%), anteverted nares (73.3%), long and smooth philtrum (73.3%), downturned corners of mouth (80.0%), high palate (80.0%), and micrognathia (73.3%) also appeared with high frequency in our cohort. Facial features and clinical characteristics of our CdLS patients are listed in Table 2. According to evaluation of growth and development, eight (53.3%) patients had intrauterine growth retardation, 14 (93.3%) showed short stature, and 14 (93.3%) had developmental delay. Skeletal malformations were mainly mild limb abnormalities, including small hands (73.3%) and 5th finger clinodactyly or short 5th finger (66.7%). A single transverse palmar crease (40.0%), 2–3 toe syndactyly (13.3%), and pectus excavatum (13.3%) were also observed. Severe limb deformity was found in only one patient (oligodactyly, 6.7%). In this cohort, the frequencies of male cryptorchidism, congenital heart disease (CHD), and renal abnormalities were 55.6%, 46.7%, and 13.3%, respectively. A total of 20.0% of patients had hearing abnormalities and 13.3% had otitis media. One patient (P2) had bilateral sensorineural deafness and received cochlear implantation at the age of 9 months. A total of 26.7% of the patients had a history of vomiting and feeding difficulty in infancy.

**Table 2:** Selected clinical data in our cohort of 15 patients with CdLS (n = 15).

Clinical findings	N/Total(%) of subjects				
	Total	*NIPBL* (11)	*SMC1A* (2)	*RAD21* (1)	*HDAC8* (1)
**Craniofacial** **features**					
Microcephaly	14 (93.3%)	11	2	0	1
Synophrys	8 (53.3%)	7	1	0	0
Highly arched eyebrow; thick eyebrow	13 (86.7%)	11	2	0	0
Long eyelashes	14 (93.3%)	11	2	1	0
Concave nasal ridge	10 (66.7%)	9	1	0	0
Anteverted nares	11 (73.3%)	10	1	0	0
Short nose	9 (60.0%)	9	0	0	0
Long philtrum; smooth philtrum	11 (73.3%)	10	1	0	0
Thin upper lip vermilion	10 (66.7%)	9	1	0	0
Downturned corners of mouth	12 (80.0%)	11	1	0	0
High palate	12 (80.0%)	9	1	1	1
Cleft palate	2 (13.3%)	1	1	0	0
Widely spaced teeth	2 (13.3%)	2	0	0	0
Micrognathia	11 (73.3%)	10	1	0	0
Ptosis	5 (33.3%)	5	0	0	0
**Growth** **abnormality**					
Intrauterine growth retardation	8 (53.3%)	7	1	0	0
Short stature	14 (93.3%)	11	1	1	1
Global developmental delay; intellectual disability	14 (93.3%)	10	2	1	1
**Musculoskeletal**					
Oligodactyly	1 (6.7%)	1	0	0	0
Small hand	11 (73.3%)	9	1	1	0
5th finger clinodactyly; short 5th finger	10 (66.7%)	8	0	1	1
2–3 toe syndactyly	2 (13.3%)	2	0	0	0
Single transverse palmar crease	6 (40.0%)	5	1	0	0
Pectus excavatum	2 (13.3%)	2	0	0	0
Hypertrichosis	3 (20.0%)	3	0	0	0
**Neurology**					
Seizures	1 (6.7%)	1	0	0	0
Abnormal muscle tone	1 (6.7%)	0	1	0	0
**Sensory** **system**					
Hearing impairment	3 (20.0%)	3	0	0	0
Otitis media	2 (13.3%)	2	0	0	0
**Malformation of the heart and great vessels**	7 (46.7%)	5	2	0	0
Atrial septal defect	3 (20.0%)	2	1	0	0
Pulmonic stenosis	3 (20.0%)	2	1	0	0
Ventricular septal defect	1 (6.7%)	1	0	0	0
Coronary-pulmonary artery fistula	1 (6.7%)	1	0	0	0
Patent ductus arteriosus	1 (6.7%)	1	0	0	0
Patent foramen ovale	1 (6.7%)	0	1	0	0
**Abnormality of the genitourinary system**	7 (46.7%)	6	1	0	0
Abnormality of kidney	2 (13.3%)	2	0	0	0
Cryptorchidism	5/9 (55.6%)	4	1	0	0
Micropenis	2/9 (22.2%)	2	0	0	0
Hypospadias	2/9 (22.2%)	2	0	0	0
**Feeding difficulties; vomiting**	4 (26.7%)	4	0	0	0

Interestingly, one patient (P10) was diagnosed with growth hormone (GH) deficiency in a local hospital. With treatment of GH, his blood glucose was as high as 43.16 mmol/L. GH treatment was then ceased and insulin treatment was instituted. After 1-week therapy, the patient received metformin treatment, and his blood glucose level was normal.

### Phenotypic comparison of our Chinese cohort with another four groups

For further understand CdLS, we did statistical analysis for the phenotype features between our cohort and a large cohort of four other groups (African and African American, Asian, Latin American, and the Middle East)^14^. As shown in Table 3, several features showed significant statistical difference in the cohorts. We found that the frequencies of synophrys, long eyelashes, short nose/anteverted nares, long philtrum, ptosis, palate anomalies, hypertrichosis, and hearing loss were significantly different among the cohorts (P values were < 0.001, 0.014, 0.028, 0.004, 0.018, 0.038, 0.001, and 0.004, respectively.). Further analysis showed that our cohort had a lower frequency of synophrys than did the African group and Latin American group. The frequency of palate anomalies in the Middle East group was higher than that in our cohort. Our cohort had a lower frequency of hypertrichosis than did the African group, Latin American group, and Middle East group. The frequency of hearing loss in the African group was higher than that in our cohort (Table 3).

**Table 3:** Clinical features of our cohort compared with four other groups.

	Our study n = 15	Africa n = 14	Asia n = 23	Latin America n = 22	Middle East n = 8	Chi-square value	p-Values
Average age (years)	4	1.4	4.3	6.5	2.6		
Age range	3m–10y2m	2w–9y	2w–12y	1d–37y	3m–8y		
*NIPBL* (%)	11/15 (73%)	6/6 (100%)	6/8 (75%)	77%	3/5 (60%)		
*HDAC8* (%)	1/15 (7%)	0	2/8 (25%)	18%	1/5 (20%)		
*SMC1A* (%)	2/15 (13%)	0	0	5%	1/5 (20%)		
*RAD21*(%)	1/15 (7%)	0	0	0	0		
Synophrys	8/15 (53.3%)	14/14 (100.0%)^a^	21/23 (91.3%)	22/22 (100.0%)^a^	7/8 (87.5%)	16.357	< 0.001
Arched eyebrows	13/15 (86.7%)	14/14 (100.0%)	21/23 (91.3%)	22/22 (100.0%)	8/8 (100.0%)	4.058	0.286
Long eyeashes	14/15 (93.3%)	14/14 (100.0%)	23/23 (100.0%)	22/22 (100.0%)	6/8 (75.0%)	7.486	0.014
Short nose/anteverted nares	11/15 (73.3%)	14/14 (100.0%)	19/23 (82.6%)	22/22 (100.0%)	7/8 (87.5%)	8.738	0.028
Long philtrum	11/15 (73.3%)	14/14 (100.0%)	23/23 (100.0%)	22/22 (100.0%)	8/8 (100.0%)	9.995	0.004
Ptosis	5/15 (33.3%)	10/14 (71.4%)	9/23 (39.1%)	8/22 (36.4%)	0/8 (0.0%)	11.612	0.018
Palate anomalies	2/15 (13.3%)	3/13 (23.1%)	2/18 (11.1%)	6/22 (27.3%)	4/5 (80.0%)^a^	9.447	0.038
Micrognathia	11/15 (73.3%)	11/13 (84.6%)	10/18 (55.6%)	13/22 (59.1%)	2/5 (40.0%)	5.011	0.285
Clinodactyly	10/15 (66.7%)	12/13 (92.3%)	10/18 (55.6%)	10/22 (45.5%)	3/5 (60.0%)	8.472	0.068
Hypertrichosis	3/15 (20.0%)	11/13 (84.6%)^a^	12/18 (66.7%)	15/22 (68.2%)^a^	5/5 (100.0%)^a^	16.713	0.001
Growth deficiency	14/15 (93.3%)	13/13 (100.0%)	18/18 (100.0%)	22/22 (100.0%)	4/5 (80.0%)	5.688	0.057
Hearing loss	1/15 (6.7%)	9/13 (69.2%)^a^	4/18 (22.2%)	7/22 (31.8%)	3/5 (60.0%)	14.644	0.004
Congenital heart disease	7/15 (46.7%)	3/13 (23.1%)	4/18 (22.2%)	9/22 (40.9%)	2/5 (40.0%)	3.478	0.500
Renal anomalies	2/15 (13.3%)	4/13 (30.8%)	2/18 (11.1%)	2/22 (9.1%)	1/5 (20.0%)	3.482	0.465
Neurologic abnormalities	2/15 (13.3%)	1/13 (7.7%)	1/18 (5.6%)	1/22 (4.5%)	0/5 (0.0%)	1.732	0.902

^a^Indicates that the incidence of phenotype was statistically significant compared with our cohort. The four other groups (African and African American, Asian, Latin American, and the Middle East) of features data were from the Dowsett et al.^14^.

## Discussion

There is a wide range of severity of clinical characteristics observed in patients with CdLS, including typical facial features, growth retardation, intellectual disability, limb defects, and involvement of other systems. These features widely vary among affected patients and range from relatively mild to severe. Facial features (synophrys, thick eyebrows and arched eyebrows, long eyelashes, anteverted nares, long and smooth philtrum, thin lips, downturned corners of the mouth, etc.) are the most clinically consistent and recognizable findings in CdLS, which suggest this syndrome in the clinic^15^. In our cohort, 11 patients (P1 ~ P11) had *NIPBL* variants, of which, 9 of these patients were diagnosed with classic CdLS (scoring > 11 points), all of them showed these facial features. The patients (P13, P14, P15) with lower score (4 points) with *SMC1A*, *RAD21* and *HDAC8* variants had only few facial features of with CdLS. Furthermore, P15 had hypertelorism and a broad nasal tip, which is consistent with other reported patients with *HDAC8* variants^16^.

In our study, most patients were referred for the chief complaint of growth retardation and developmental delay, which are the common features of most CdLS patients. Based on the assessment of growth and development, fourteen (93.3%) patients had developmental delay, 53.3% of the patients had intrauterine growth retardation and 93.3% of the patients showed short stature (60.0% of these patients had a height below—3SD). Skeletal anomalies ranged from small hands to more severe reduction defects of the fingers. Small hands and 5th finger clinodactyly were the most common anomalies in all of our patients. Additionally, a single transverse palmar crease, 2–3 toe syndactyly, and pectus excavatum were observed in our patients. Other system disorders are also involved in CdLS. Feeding problems are typical in infancy in CdLS. In our cohort, four (26.7%) patients had feeding difficulties and vomiting. The incidence of CHD in CdLS is reported to approximately 14–70%, and the most common CHDs are pulmonic and peripheral pulmonic stenosis, followed by ventricular septal defect and atrial septal defect^17^. In our cohort, the incidence of CHD was 46.7%, with mainly pulmonic stenosis and atrial septal defect. Cryptorchidism was commonly found in our male patients. Interestingly, hyperglycemia was also observed in one patient (P10). Type 2 diabetes mellitus develops in 4% of individuals in adulthood^18^. However, there is no clear evidence of an increased risk of diabetes in children with CdLS, and no other similar cases have been published. Therefore, hyperglycemia probably occurred with CdLS in our patient by chance.

Several features (synophrys, palate anomalies, hypertrichosis, and hearing loss) in our cohort were significantly different from four other groups (African and African American, Asian, Latin American, and the Middle East^14^). We speculate that the low incidence of synophrys and hypertrichosis in our cohort compared with other groups may be a result of ethnic differences in hair density. With regard to involvement of other systems, CHD, renal anomalies, and neurological abnormalities were not significantly different between our cohort and four other groups. Hearing loss had a lower frequency in our cohort than in the African group which may be due to the different gene variation types. In the African group, all patients had *NIPBL* gene variants. In the future, we hope to enroll more cases in order to evaluate the differences between phenotypic findings between our cohort and other cohorts.

CdLS is characterized by a wide genetic heterogeneity and caused by cohesin complex-associated genetic variants. The most commonly known genetic cause of CdLS is *NIPBL* gene variants, which can be identified in approximately 70% of cases^2^. The *NIPBL* gene is located on chromosome 5p13.2, and it spans more than 190 kb and contains 47 exons. To date, more than 300 different *NIPBL* variants have been reported in patients with CdLS, including missense/nonsense, splicing, and regulatory variants, and deletions and insertions. In our study, 11 *NIPBL* variants were identified, among which c.6109-1G > A (P1), c.6763 + 5G > T (P2), c.7264-6T > G (P3), and c.-79-2A > G (P4) caused a disease phenotype by breaking the wild-type splice acceptor site of the *NIPBL* gene, which led to formation of alternative transcripts by aberrant splicing. Nucleotide transition in c.5683A > G (P5), 5615T > A (P6), and c.6722T > C (P7) led to substitution of normal residues, which is susceptible to forming abnormal protein structures. Moreover, c.6854_6855delAG (P8), c.330_331delAA (P9), c.3344G > A (P10), and c.4310T > G (P11) resulted in a premature stop codon. Except for the variant in P8, the other 10 variants appear to be de novo variants in the patients because they were absent in their parents. Variants in *SMC1A* residing at Xp11.22 account for approximately 5% of individuals^19^. P12 had a missense variant [p. (Cys781Phe)] in exon 15 of the *SMC1A* gene. And sequencing results showed that his mother carried the same variant, while the mother had no special phenotype. A missense variant was identified in P13 [p. (Arg363Ile)] and this was novel. Variants in *RAD21* (8q24.11) and *HDAC8* (Xq13.1) have been described in only a few patients. P14 had a shift frame deletion secondary to a variant of c.1553_1554delAG in exon 12 of the *RAD21* gene, which led to a premature stop codon. A novel splicing variant of *HDAC8* (c.628 + 1G > C) was identified in P15.

Studies that have reported genotype–phenotype correlations in CdLS have described variability in clinical characteristics within and between variants. Patients with *NIPBL* variants are likely to present with more severe clinical features and to have more impaired cognitive function than those with other causal variants^20^. In our study, P13 with an *SMC1A* variant, P14 with an *RAD21* variant, and P15 with an *HDAC8* variant had non-typical facial features and mild phenotype. However, compared with the previously reported patients, P12 who had an *SMC1A* variant had more severe phenotype, including CHD, cleft palate and typical facial features. Therefore, the phenotype varies even for unrelated patients with the same variant, suggesting other genetic or environmental modifying factors. Furthermore, a truncated and presumably nonfunctional NIPBL protein caused by variants (nonsense, splice site, and frame shift variants) is usually associated with a more severe cognitive and structural phenotype than missense variants. However, missense variants associated with the HEAT domain cause severe phenotype^5^. In our study, P7 who had an *NIPBL* missense variant had a severe phenotype with oligodactyly, severe developmental delay, and growth retardation. This finding supports the notion that variants affecting the HEAT domain play a critical role in protein function. The patients with a non classical CdLS phenotype in this cohort (P14, P15) mainly presented with growth and developmental delay, 5th finger clinodactyly and short 5th finger and the molecular testing was needed to confirm the diagnosis. The extensive phenotypic and genetic heterogeneity of cohesinopathies difficults the diagnosis. Growth retardation and intellectual disability might be the main clinical manifestations in patients with mild facial phenotype.

At present, clinical interventions for patients with CdLS are mainly symptomatic treatment. Additionally, rehabilitation training appears to be a good option for improving motor development. A previous report indicated that GH therapy may be an effective method to improve the height of patients^21^. However, the benefits of increased growth by GH supplementation should be weighed against the burden of daily subcutaneous injections and the lack of a positive impact of an increased adult height on the quality of life for most individuals with CdLS, as indicated in Kline AD et al. 2018^2^. Thus, GH therapy and growth curves require further investigation in the future.

## Conclusion

We analyzed 15 Chinese cases of CdLS secondary to *NIPBL*, *SMC1A,* *RAD21,* or *HDAC8* variants, and among them, 11 were novel. Variants in the *NIPBL* gene were the most common cause in our cohort. Furthermore, presentations vary in children with CdLS. Clinical manifestations of patients in our cohort are mostly consistent with other ethnicities, but several clinical features have different frequencies. There are also some limitations of our study. This study was performed in one institute, which may have created a selection bias. Future research should expand the survey sample. And caution needs to be considered while interpreting the results, even though the results will be useful to explore the spectrum of CdLS, functional significance of the identified variants is needed.

## Supplementary information

Supplementary Information.

## Abbreviations

ACMG: American College of Medical Genetics and Genomics
ASD: Atrial septal defect
CA-PAF: Coronary-pulmonary artery fistula
CdLS: Cornelia de Lange syndrome
CHD: Congenital heart disease
GH: Growth hormone
HGMD: Human gene mutation database
HPO: Human phenotype ontology
NGS: Targeted next-generation sequencing
PDA: Patent ductus arteriosus
PFO: Patent foramen ovale
PS: Pulmonic stenosis
SD: Standard deviation
VSD: Ventricular septal defect
