# Supplementary material for: Clinical and molecular analysis in a cohort of Chinese children with Cornelia de Lange syndrome
Source: Sci Rep. 2020 Dec 4;10:21224. doi: 10.1038/s41598-020-78205-5 (PMC7718889; doi:10.1038/s41598-020-78205-5)
Supplement: Supplementary file 1 — Supplementary Information. [file 41598_2020_78205_MOESM1_ESM.doc]

**Title page**

**Clinical and molecular analysis in a cohort of Chinese children with** **Cornelia de Lange syndrome**

Qun Li^1, #^ (liqun9201@126.com), Guoying Chang^1,#^ (changguoying@126.com), Lei Yin^2^ (scmc1678@sina.cn), Juan Li^1^ (ljuan_scmc@126.com), Xiaodong Huang^1^ (jcperkesh@126.com), Yongnian Shen^1^ (syn14401@hotmail.com), Guoqiang Li^3^ (18817557506@126.com), Yufei Xu^3^ (390948063@qq.com), Jian Wang^3, *^ (labwangjian@shsmu.edu.cn), Xiumin Wang^1,*^(wangxiumin1019@126.com)

^1^ Department of Endocrinology and Metabolism, Shanghai Children’s Medical Center, Shanghai Jiaotong University School of Medicine, Shanghai, China

^2^ Department of Rare Disease Clinic, Shanghai Children’s Medical Center, Shanghai Jiaotong University School of Medicine, Shanghai, China

^3^ Department of Medical Genetics and Molecular Diagnostic Laboratory, Shanghai Children’s Medical Center, Shanghai Jiaotong University School of Medicine, Shanghai, China

^#^ These authors contributed equally to this work, and should be considered as co-first authors. Qun Li (liqun9201@126.com), Guoying Chang (changguoying@126.com).

^*^ Corresponding author: Dr. Jian Wang (labwangjian@shsmu.edu.cn) and Dr. Xiumin Wang (wangxiumin1019@126.com), Shanghai Children’s Medical Center, Shanghai Jiaotong University School of Medicine. 1678 Dongfang Road, Shanghai 200127, China; Phone number: 38626161

**Supplementary data**

**Supplementary Table S1. Classification of pathogenicity based on the ACMG guideline**

| **Case** | **Variants** | **Pathogenic** | | | | **Classifi-cation** |
| --- | --- | --- | --- | --- | --- | --- |
|  |  | **very strong** | **strong** | **moderate** | **supporting** |  |
| 1 | *NIPBL* c.6109-1G>A | PVS1 | PS2 | PM2 | / | P |
| 2 | *NIPBL* c.6763+5G>T | / | PS1+PS2 | PM2 | PP3 | P |
| 3 | *NIPBL* c.7264-6T>G | / | PS2 | PM2 | PP3 | LP |
| 4 | *NIPBL* c.-79-2A>G | / | PS2 | PM2 | / | LP |
| 5 | *NIPBL* c.5683A>G, p. (Arg1895Gly) | / | PS2 | PM1+PM2+PM5 | PP3 | P |
| 6 | *NIPBL* c.5615T>A, p. (Leu1872His) | / | PS2 | PM1+PM2 | PP3 | LP |
| 7 | *NIPBL* c. 6722T>C, p. (Leu2241Pro) | / | PS2 | PM1+PM2 | PP3 | LP |
| 8 | *NIPBL* c.6854_6855delAG, p. (Gln2285Argfs*3) | PVS1 | / | PM2 | / | LP |
| 9 | *NIPBL* c.330_331delAA, p. (Ser111Hisfs*16) | PVS1 | PS2 | PM2 | / | P |
| 10 | *NIPBL* c.3344G>A, p. (Trp1115*) | PVS1 | PS2 | PM2 | / | P |
| 11 | *NIPBL* c.4310T > G, p. (Leu1437*) | PVS1 | PS2 | PM2 | / | P |
| 12 | *SMC1A* c.2342G>A, p. (Cys781Phe) | / | PS1 | PM1+PM2 | PP3 | LP |
| 13 | *SMC1A* c.1088G>T, p. (Arg363Ile) | / | PS2 | PM2 | PP3 | LP |
| 14 | *RAD21*c.1553_1554delAG, p. (Glu518Valfs*18) | PVS1 | / | PM2 | / | LP |
| 15 | *HDAC8* c.628+1G>C | PSV1 | PS2 | PM2 | / | P |
| PVS: pathogenic very strong; PS: pathogenic strong; PM: pathogenic moderate; PP: pathogenic supporting; P: pathogenic; LP: likely pathogenic | | | | | | |

**Supplementary Table S2**. Summary of clinical characteristics of 15 patients with CdLS

| **Patient ID** | **1** | **2** | **3** | **4** | **5** | **6** | **7** | **8** |
| --- | --- | --- | --- | --- | --- | --- | --- | --- |
| Gender | F | F | M | F | M | M | F | M |
| Gestation (weeks) | Full term | Full term | Full term | Full term | Full term | Full term | Full term | 35 |
| Birth weight (kg) | 2.13 | 2.44 | 2.9 | 2.7 | 2.45 | 2.0 | 1.45 | 2.1 (P10~P50) |
| Age | 3m | 3y8m | 4y5m | 4y | 6m | 2y2m | 4y4m | 1y5m |
| height (cm) | 48  (-5.81SD) | 85  (-4.26SD) | 92  (-3.79SD) | 92  (-2.92SD) | 63  (-2.30SD) | 75.5  (-4.24SD) | 85  (-5.29SD) | 64  (-6.00SD) |
| weight (kg) | 3.0  (-5.70SD) | 11.5  (-2.74SD) | 13.0  (-2.73SD) | 12.4  (-2.40SD) | 7.6  (-0.90SD) | 8.8  (-3.50SD) | 8.5  (-5.60SD) | 6.0  (-5.14SD) |
| **Growth abnormality** |  |  |  |  |  |  |  |  |
| Intrauterine growth retardation (HPO:0001511) | + | + | - | - | + | + | + | - |
| Short stature (HPO:0004322) | + | + | + | + | + | + | + | + |
| **Craniofacial features** |  |  |  |  |  |  |  |  |
| Microcephaly (HPO:0000252) | + | + | + | + | + | + | + | + |
| Synophrys (HPO:0000664) | + | - | + | + | + | - | - | + |
| Highly arched eyebrow; Thick eyebrow (HPO:0002553; HPO:0000574) | + | + | + | + | + | + | + | + |
| Long eyelashes (HPO:0000527) | + | + | + | + | + | + | + | + |
| Concave nasal ridge (HPO:0011120) | + | + | - | + | + | + | + | + |
| Anteverted nares (HPO:0000463) | + | + | + | + | + | + | + | + |
| Short nose (HPO:0003196) | + | + | + | + | + | - | + | + |
| Long philtrum; Smooth philtrum (HPO:0000343; HPO:0000319) | + | + | + | + | + | + | + | + |
| Thin upper lip vermilion (HPO:0000219) | + | + | + | + | + | - | + | + |
| Downturned corners of mouth (HPO:0002714) | + | + | + | + | + | + | + | + |
| High palate (HPO:0000218) | + | + | - | + | + | + | + | + |
| Cleft palate (HPO:0000175) | - | - | - | - | - | - | - | - |
| Widely spaced teeth (HPO:0000687) | - | - | - | - | - | - | + | - |
| Micrognathia (HPO:0000347) | + | + | + | + | + | + | + | + |
| Ptosis (HPO:0000508) | - | - | + | - | - | + | + | - |
| **Musculoskeletal** |  |  |  |  |  |  |  |  |
| Oligodactyly (HPO:0012165) | - | - | - | - | - | - | + | - |
| Small hand (HPO:0200055) | + | + | - | + | + | + | + | + |
| 5th finger clinodactyly; Short 5th finger (HPO:0004209; HPO:0009237) | + | + | - | + | + | - | + | + |
| 2-3 toe syndactyly  (HPO:0004691) | - | - | - | - | + | - | - | - |
| Single transverse palmar crease (HPO:0000954) | + | + | - | - | - | - | - | + |
| Pectus excavatum  (HPO: 0000767) | + | - | - | - | - | - | - | - |
| Hypertrichosis (HPO:0000998) | - | - | - | - | - | - | - | - |
| Others | index finger deformity | - | - | - | - | - | - | - |
| **Global developmental delay; Intellectual disability (HPO:0001263; HPO:0001249)** | + | + | - | + | + | + | + | + |
| **Other major systems** |  |  |  |  |  |  |  |  |
| **Neurology** |  |  |  |  |  |  |  |  |
| Seizures (HPO:0001250) | - | - | - | - | - | + | - | - |
| Abnormal muscle tone (HPO:0003808) | - | - | - | - | - | - | - | - |
| **Sensory system** |  |  |  |  |  |  |  |  |
| Hearing impairment (HPO:0000365) | - | + | - | - | - | - | - | - |
| Otitis media (HPO:0000388) | - | - | - | - | + | - | - | - |
| **Malformation of the heart and great vessels (HPO:0002564)** | PS, ASD | - | - | - | - | - | ASD | PS, VSD |
| **Abnormality of the genitourinary system (HPO:0000119)** | - | - | Cryptorchidism | - | Hypospadias | Horseshoe kidney,  renal cyst | - | Cryptorchidism |
| **Feeding difficulties; Vomiting (HPO:0011968; HPO:0002013)** | + | - | - | - | - | - | + | - |
| **Clinical score** | 14 | 14 | 10 | 13 | 14 | 12 | 16 | 13 |
| **Gene variants** | *NIPBL* | *NIPBL* | *NIPBL* | *NIPBL* | *NIPBL* | *NIPBL* | *NIPBL* | *NIPBL* |
| **Patient ID** | **9** | **10** | **11** | **12** | **13** | **14** | **15** |  |
| Gender | F | M | M | M | M | M | F | 9M/6F |
| Gestation (weeks) | Full term | Full term | Full term | 36 | Full term | Full term | Full term |  |
| Birth weight (kg) | 2.3 | 3.0 | 1.8 | 2.05 (<P10) | 3.4 | 2.9 | 2.7 |  |
| Age | 2y5m | 10y2m | 3y7m | 5y2m | 4y | 5y | 5y2m |  |
| Current height (cm) | 73  (-5.29SD) | 128  (-2.10SD) | 80  (-5.65SD) | 102  (-2.44SD) | 98  (-1.56SD) | 95  (-3.90SD) | 99.1  (-2.92SD) |  |
| Current weight (kg) | 7.0  (-5.30SD) | 26.4  (-1.43SD) | 7.5  (-6.08SD) | 17.0  (-1.11SD) | 15  (-0.93SD) | 18.0  (-0.46SD) | 14  (-2.44SD) |  |
| **Growth abnormality** |  |  |  |  |  |  |  |  |
| Intrauterine growth retardation  (HPO:0001511) | + | - | + | + | - | - | - | 8/15 (53.3%) |
| Short stature (HPO:0004322) | + | + | + | + | - | + | + | 14/15 (93.3%) |
| **Craniofacial features** |  |  |  |  |  |  |  |  |
| Microcephaly (HPO:0000252) | + | + | + | + | + | - | + | 14/15 (93.3%) |
| Synophrys (HPO:0000664) | + | - | + | + | - | - | - | 8/15 (53.3%) |
| Highly arched eyebrow; Thick eyebrow (HPO:0002553; HPO:0000574) | + | + | + | + | + | - | - | 13/15 (86.7%) |
| Long eyelashes   (HPO:0000527) | + | + | + | + | + | + | - | 14/15 (93.3%) |
| Concave nasal ridge (HPO:0011120) | + | - | + | + | - | - | - | 10/15 (66.7%) |
| Anteverted nares (HPO:0000463) | + | - | + | + | - | - | - | 11/15 (73.3%) |
| Short nose (HPO:0003196) | + | - | + | - | - | - | - | 9/15 (60.0%) |
| Long philtrum;  Smooth philtrum  (HPO:0000343; HPO:0000319) | + | - | + | + | - | - | - | 11/15 (73.3%) |
| Thin upper lip vermilion (HPO:0000219) | + | - | + | + | - | - | - | 10/15 (66.7%) |
| Downturned corners of mouth (HPO:0002714) | + | + | + | + | - | - | - | 12/15 (80.0%) |
| High palate (HPO:0000218) | + | + | - | + | - | + | + | 12/15 (80.0%) |
| Cleft palate (HPO:0000175) | + | - | - | + | - | - | - | 2/15 (13.3%) |
| Widely spaced teeth (HPO:0000687) | - | + | - | - | - | - | - | 2/15 (13.3%) |
| Micrognathia (HPO:0000347) | + | - | + | + | - | - | - | 11/15 (73.3%) |
| Ptosis (HPO:0000508) | + | - | + | - | - | - | - | 5/15 (33.3%) |
| **Musculoskeletal** |  |  |  |  |  |  |  |  |
| Oligodactyly (HPO:0012165) | - | - | - | - | - | - | - | 1/15 (6.7%) |
| Small hand (HPO:0200055) | + | - | + | + | - | + | - | 11/15 (73.3%) |
| 5th finger clinodactyly; Short 5th finger (HPO:0004209; HPO:0009237) | + | - | + | - | - | + | + | 10/15 (66.7%) |
| 2-3 toe syndactyly (HPO:0004691) | - | - | + | - | - | - | - | 2/15 (13.3%) |
| Single transverse palmar crease (HPO:0000954) | + | - | + | - | + | - | - | 6/15 (40.0%) |
| Pectus excavatum  (HPO: 0000767) | - | + | - | - | - | - | - | 2/15(13.3%) |
| Hypertrichosis   (HPO:0000998) | + | + | + | - | - | - | - | 3/15 (20.0%) |
| Others | - | - | - | - | - | - | Hypertelorism  broad nasal tip |  |
| **Global developmental delay; Intellectual disability (HPO:0001263; HPO:0001249)** | + | + | + | + | + | + | + | 14/15 (93.3%) |
| **Other major systems** |  |  |  |  |  |  |  |  |
| **Neurology** |  |  |  |  |  |  |  |  |
| Seizures (HPO:0001250) | - | - | - | - | - | - | - | 1/15 (6.7%) |
| Abnormal muscle tone (HPO:0003808) | - | - | - | + | - | - | - | 1/15 (6.7%) |
| **Sensory system** |  |  |  |  |  |  |  |  |
| Hearing impairment (HPO:0000365) | + | - | + | - | - | - | - | 3/15 (20.0%) |
| Otitis media (HPO:0000388) | + | - | - | - | - | - | - | 2/15 (13.3%) |
| **Malformation of the heart and great vessels (HPO:0002564)** | - | CA-PAF | PDA | PS, ASD | PFO | - | - | 7/15 (46.7%) |
| **Abnormality of the genitourinary system**  **(HPO:0000119)** | - | Small kidney,  Cryptorchidism,Micropenis | Cryptorchidism,  Micropenis,  Hypospadias | Cryptorchidism | - | - | - | 7/15 (46.7%) |
| **Feeding difficulties;** **Vomiting (HPO:0011968; HPO:0002013)** | + | - | + | - | - | - | - | 4/15 (26.7%) |
| **Clinical score** | 15 | 8 | 15 | 13 | 4 | 4 | 4 |  |
| **Gene variants** | *NIPBL* | *NIPBL* | *NIPBL* | ***SMC1A*** | ***SMC1A*** | ***RAD21*** | ***HDAC8*** |  |
| F: female; M: male; SD: standard deviation; HPO: human phenotype ontology; PS: pulmonic stenosis; VSD: ventricular septal defect; ASD: atrial septal defect; CA-PAF: coronary-pulmonary artery fistula; PDA: patent ductus arteriosus; PFO: patent foramen ovale | | | | | | | | |
